# 

**Published:** 1876-05

**Authors:** 


### THE AMERICAN JOURNAL OF DENTAL SCIENCE.

EDITED BY

F. J. S. GORGAS, M. D., D. D. S.

VOL. 10.—THIRD SERIES.

SURGEON GEN'LS OFFICE
73810
LIBRARY.

BALTIMORE:

SNOWDEN & COWMAN.

LONDON:

TRUBNER & CO., 60 PATERNOSTER ROW.

1876—1877.

### INDEX TO VOLUME X.---THIRD SERIES.

A New Dental Hospital, - - - - - 192
A New Medical Journal, - - - - - 572
A Sensible Precaution, - - - - - 432
A Strange Case, - - - - - - 562
A Fatal Case of Parotitis, - - - - - 523
A Dangerous Dentist, - - - - - 41
A Portion of a Tooth Imbedded in Lower Lip, - 18
A Large Mouth, - - - - - - 96
Absorption of Dentine, - - - - - 278
Address before Society of Alumni of Baltimore College of Dental Surgery, - - - - - 529
An Effectual Method of applying Iodine to Cysts, - 138
An Extraordinary Story, - - - - - 141
Advance in Dental Surgery, - - - - 380
Alumni Association of Baltimore College of Dental Surgery, 541
Amalgam Question, - - - - - - 433
American Academy of Dental Science, 115, 169, 211, 312
American Academy of Dental Surgery, - 310, 438, 481
American Dental Association, Notes on the - 1
American Dental Association, - - 170, 193
American Dental Convention, - - - - 170
Anæsthesia of Drunkenness Recommended, - - 190
Anatomical and Physiological Differences between the White and Negro Races, - - - 289
Alveolar Abscess, - - - - - - 443
Antiseptic Property of Borax, - - - - 240
Air as an Anæsthetic, - - - - - 23
Artistic Knowledge and Critical Taste in Dentistry, - 7
Artificial Separation of the Teeth, - - 67
Arrest of Hemorrhage by Torsion, - - - - 88
Alumni Notice, - - - - - - - 471

Allen, John, D. D. S., - - - - 485
Acid Conditions of the Fluid of the Mouth, - - 521
Anæsthesia of Ether and Chloroform, Differences between, 378
Atropia, Local Action of, - - - - 431
Atkinson, W. H., M. D., D. D. S , - - - 318
Avoidance of Phosphorous Poisoning, - - - 45
Baltimore College of Dental Surgery—Thirty-Seventh Annual Commencement. - - - 548
Bacteria and Fermentation, - - - - 555
Barrett, W. C., D. D. S, - - - 552, 12
Bonwill, W. G. A., D. D. S., - - - 23
Bonwill's Method of Inducing Anæsthesia, - 87, 472
Boracic Acid and its application, - - - 316
Boracic Acid, - - - - - 189
Boracic Acid for Ringworm, - - - - 188
Borax as an Antiseptic, - - - - 45
Borax, Antiseptic Property of, - - - 240
Bibliographical.
Wilkerson's Dental Register and Examiner, - 282
Micro-Photographs in Histology, - 282, 476
A Manual of Dental Anatomy, - - 376
A History of Dental and Oral Science in America, 377
The Scientific American, - - - 473
The Students' Guide to Dental Anatomy and Surgery, 474
The American Agriculturist, - - - 475
Physicians Visiting List for 1877, - - 475
Vick's Floral Guide for 1877, - - - 477
The Pen and Plow, - - - - - 477
Black, G. V., D. D. S., - - - - 225
Burt, W. J., M. D.. - - - - - 289
California State Dental Association, - - - 63
Carbolic Acid Poisoning, - - - - 238
Carbolic Acid as a Local Anæsthetic, - - 432
Callender, G. W., M. D., - - - - 417
Carbolic Acid Sprays in Throat Affections, - 142
Caution in the Use of Chloral, - - - 284
Celluloid, - - - - - - - 122, 545
Chemistry of Salicylic Acid, - - - - 336
Chase, H. S., D. D. S., M. D., - - 84, 171, 419
Chloral Plaster in Neuralgia, - - - 429, 524

Chloral as an Anæsthetic, - - - 42
Chloroform. - - - - - - 576
Chittenden, C. C., D. D. S., - - - - 110
Cooking by Cold, - - - - - 92
Cosmoline, - - - - - - 48
Correction, - - - - - - - 280
Composition of the Human Body, - - - 285
Cement for Rubber and Glass, - - - - - 282
Congenital Cleft Palate, - - - - 429
Chloroform and Dentistry, - - - - 464
Chemical and Porcelain, - - - - 522
Clark, Lane, D. D. S., - - - - - 422
Chemical Remarks on Cases of Acute Necrosis, - 414
Cremation as a Science, - - - 480
Crystal Gold, - - - - - 304
Cravens, J. D., D. D S., - - - 28, 158, 274
Cutler, S. P., M. D., D. D. S., - - - 1, 85, 337
Choice for Sedative for Young and Old, - - 524
Chromic Acid for Warts, - - - - 239
Cold Water in Neuralgia, - - - - 526
Cure for Corns, - - - - - - - 96
Cure for Tetanus, - - - - - 426
Cylinder Filling, - - - - - 79
Dangers of Breathing by the Mouth, - - - 192
Dental Education, - - - - - 515
Dental Caries, - - - - - - 35
Dental Pulp, Treatment of, - - - - 422
Dental Education, - - - - - - 12
Dental Decay, Pressure and Contact as Cause of, - 171
Dental Exhibit at the Centennial, - - - 329
Dental College Education, - - - - 250
Dental Therapeutics, - - - - - 105
Dentine, - - - - - 558
Degeneration of the Race, - - - - - 74
District Dental Society, - - - - - 66
Diseased Germs, - - - - - 185
Death from Boxing of the Ears, - - - 333
Decay of the Teeth, Cause of, - - - 334
Death from Chloroform, - - - - 335, 431, 518
Death from Ether, - - - - 383, 426

Dressing for Burns, - - - - - 189
Effects of Violent Pain on Animals and Men, - 527
Ether and Chloroform, - - - - - - 34
Extracting Teeth for Brain Disease, - - - 47
Electricity in Dental Surgery, - - - - 62
Electro-Magnetism as a Motive Power, - - 240
Epithelioma of Face, - - - - - 373
Epulis of Lower Jaw, - - - - 132
Eucalyptus in Ague, - - - - 96
European Visitor, - - - - - 426
Excision of Nerves for Spasmodic Contraction of Muscles,. 528
Exposure of Spiritualism, - - - - 94
Exercise for the Teeth, - - - - - 520
Eye-Sight, - - - - - - - 143
Fagge, C. Hilton, M. D., - - - - 183
Female Doctors in National Association, - - 190
Female Medical Students, - - - - 89
Female Students, - - - - - 46
Fetid Feet and Arms, Lotion for, - - - 95
Female Doctors in Europe, - - - - 335
Female Doctors, - - - - - - 288
Finishing Fillings, - - - - - 360
Filling Poots, A New Way of, - - - 84
For Burns, - - - - - - - 48
Food for Lean Women, - - - - 92
Fracture, A Curious Case of, - - - - 89
Fracture of Maxillae, - - - - 238
Fractured Teeth, Treatment of, - - - 375
Function of the Uvula, - - - - 479
Gallium, The New Metal, - - - 186
Gelseminum Sempervirens in Facial Neuralgia, - 95
Georgia State Dental Society, - - - 17
Glycerine, Poisonous Properties of - - - 384
Graduates in Dentistry, - - - - - 96
Hardening Compound for Steel, - - - 93
Haskell, Dr. L. P., - - - - - - 97
Hodson, J. F., D. D. S., - - - - 31
Henkel, S. H., D. D. S., - - - - 105
Help for the Deserving, - - - - 136
Headache from Eye-Strain, - - - 380

How best to Establish a Dental Practice, - - 400
How to Cure a Cold in the Head, - - 143
Hodgkin, Prof. James B., D. D. S., - - 385, 433
Horne, W. C., D. D. S., - - - 179
How do Nerves Transmit Impulses, - - 188
How to Check Coughs, - - - 188
Hunt, C. P., Dr., - - - - 439
Hygienic Laws, - - - - 241
Improvements in Dentistry, - - - 225
Inflammatory process—Its Nature, - - 381
Influence of Anæsthetics on the Sexual Impressions of Females, - - - - 479
Influence of Age of Mother on Sex, - - 582
Is Alcohol a Food ? - - - 427, 480
Is Dental Surgery holding its own ? - - - 165
Jaborandi in Colds. - - - - 93
Johns Hopkins Hospital, - - - 96
Johnston, Prof. Christopher, M. D., - - 145
Judd, H., M. D., D. D. S., - - - 360
Klump, G. W., D. D. S., - - - - 241
Local Action of the So-called Astringents on the Blood Vessels, - - - - 421
Local Anæsthesia, New Mode of Obtaining, - 90
Lead Line on Gums, - - - 183, 286
Lupus of the Mouth and Pharynx, - - 564
Maryland Dental College, Fourth Annual Commencement, 551
Mason, Francis, F. R., C. S., - - - 18
Mechanical Dentistry, - - - - 97
Mastic Chewing, - - - - - 432
Medical Women, - - - - - 428
Medicated Ice, - - - - - 47
Means to Make Leeches Take Immediately, - 336
Mears, J. Ewing, M. D., - - 263, 297
Mercurial Poisoning from Canned Meat, - - 384
Medical Diploma for Women, - - 192
Methods of, and Materials For Filling Teeth, - 216
Milk as a Vehicle of Contagion, - - 44
Morphia in Neuralgia, - - - - 91
Murray, John, D. D. S., - - - 74
Natural Ink, - - - - 143

Necrosis of the Lower Jaw, - - - 45
Neuralgia, Treatment of, - - - 191
Nerve Injury, The result of, - - - 566
New York Odontological Society, - 389, 495, 456
New York State Dental Society, - - 115
Nervous Headache and Neuralgia, - - - 114
Nitrite of Amly. - - - - 142
Nervous Sensitiveness, - - - - 525
Nerve Stretching in Tetanus, - - - 287
Nervous Exhaustion, - - - - - 323
North Carolina State Dental Association, - - 170
OBITUARY.
Prof. Elias Wildman, M. D., D. D. S., - 236
Obliteration of Deformity from Depressed Cicatrices, 380 472
Outline of Secture, - - - - 385
On the Profession of Dental Surgery, - - 366
On Dentine, - - - - - 558
Odontological Society of Great Britain, - - 267
Painful Points in Neuralgia, - - - 93
Painless Opening of Abscesses, - - - 42
Perine, George H., D. D. S., - - - 7, 62
Palmer, Dr. Edgar, - - - - - 165
Partial Exsection of both Upper Jaws for Enchondroma, 572
Phases of Professional Development, - - 179
Phosphate of Lime as a Therapeutic Agent, - 567
Physiology of the Nerve Centres, - - - 379
Physiological Action of Alcohol, - - - 374
Physical History of Various Nations of the Earth with
Special Reference to their Teeth, - - 485
Pollock, G. D., F. R., C. S., - - - 443
Plastic Filling, - - - - - 337
Poisonous Properties of Glycerine, - - 384
Prevention and Treatment of Approximate Decay, - 257
Professional Inventions, - - - - 571
Progress of Cadaveric Decay, - - - 288
Proportion of Medical Men to Population in England, 190
Psoriasis of the Tougue, - - - - 567
Pulp Capping, - - - - - - 552
Radical Cure for Piles, - - - - 93
Reform in Medical Colleges, - - - 135

Resection of a Portion of Lower Jaw, - - 43
Repairing Thermometers, - - - - 478
Resetting and Transplanting Teeth, - - 344, 354
Report on Surgery, - - - - 145
Relation of Alcohol to Animal Heat and Muscular Power, 190
Salicylic Acid for Offensive Breath, etc., - - 91
Salicylic Acid, Chemistry of - - - 336
Salicylic Acid, External Use of, - - - 410
Salicylic Acid, Poisonous Effects of, - - 527
Salicylic Acid, Irritating Effects of, - - - 430
Sarcomatous Tumors of the Jaw, - - - 263
Scott, J. Clark, D. D. S., - - - - 562
Sensitive Artificial Eyes, - - - - 95
Sibler, Chas., M. D., - - - - 558
Soft Hands, - - - - - 144
Southern Dental Association, - - - 570
Some Health Principles, - - - - 139
Skull Changes in Rickets, - - - - 90
Spalding, C. W., D. D. S., - - - - 79
Steatomatous Tumor, Removal of, - - - 327
Sulphide of Calcium, - - - - 383
Talbot, E. S., D. D. S., - - - - 250
Taft, William, D. D. S., - - - 344
Thackston, W. W. H , M. D., D. D. S., - - 529
Tomes, John, F. R. S., - - - - 366
Transplantation and Replantation of Teeth, - 344, 354
The Air as an Anæsthetic, - - - - 23
The Blue Line in Lead Poisoning, - - - 43
Tumors of the Jaws, - - - - 263
The Discovery of Anæsthesia, - - - 46
The Effects of Medicines upon the Teeth, - - 31
The Eyes and the Teeth, Have they any Nervous Connection? 28
The Old and the New, - - - - 419
Treatment of Abscesses by Hyper-Dentition with Carbolized
Water, - - - - - 417
The Food we Eat, - - - - - 39
The Dentist's Future, - - - 470
The Toxic Properties of Glycerine, - - - 479
Tooth Paste, - - - - - - 478
To the Dental Profession, - - - - 467
The Parasitic Theory of Diseases, - - 90

The Stuck Method Simplified, - - - - 37
Tincture of Aconite Root for Odontalgia, - - 528
The Lost Arts, - - - - - - 519
Treatment of Teeth during Gestation and Lactation, 510
The Teeth and their Preservation, - - - 49
To Check Epistaxis, - - - - - 46
Topical Applications in Painful Dentitions, - 44
Torsion on Arteries, - - - - 93
Treatment of Exposed Pulps, - - - - 110
Toothache, - - - - - - 116
Treatment of Convulsions in Infants, - - 133
The Plethysmograph, - - - - - 48
The Use of Pestles and Mortars, - - - 38
Treatment of Cysts, - - - - - 140
Treatment of Children in Egypt, - - - 140
Treatment of Stammering, - - - - - 141
Thomas, G. R., - - - - . 67
Tooth Anomaly, - - - - 8
Treatment of Exposed Pulps by Lacto-Phosphate of Lime, 274
The Blue Line in Lead Poisoning, - - - 286
The Effects of Ether and Chloroform, - - 283
Toothache Remedy, - - - 189, 286
Tees, Ambler, D. D. S., - - - - - 304
Tumors and Abscesses, - - - - - - 318
The Recent Meeting of American Dental Association, 233
The Centennial, - - - - - - 235
To Cut Cold Steel with Soft Iron, - - - - 237
The Cuca or Coca Leaf and its Effects, - - 187
Toothache, - - - - - - - 189
The Skin and Bodily Temperature, - - 191
Ulceration of Fraenum Linguae, - - - - 381
Ununited Fracture, Treatment of - - - - 574
Valuable Mouth Washes, - - - 478
Vegetable Diet in Relation to Longevity, - - 141
Venous Pulse an Habitual Symptom of the Physiological
Action of Chloroform, - - - 576
Value of Phosphate of Lime as a Therapeutic Agent, 370
Virginia State Dental Association, - - 328, 408
Vulcanite, - - - - - - - 424
Washington s Teeth, - - - - - 127
Wadsworth, H. N., D. D. S., - - - - - 354
Warner, A, Jr., D. D. S., - - - - 257
Washing and Pressing of Amalgams, - - 372
Webb, M. H., D. D. S., - - - - - 49
Wheeler, Dr. E. G , - - - - - - 400
Williams, J., D. D. S., - - - - - 510
Wingate, John D., D. D. S., - - - - - 37
Wheat and Flour, - - - - - 280
Wounds, - - - - - - 144
Women's Sphere, - - - - - 286

*Georgia State Dental Society.*

The Eighth Annual Meeting of this Society will be held in Atlanta, Georgia, May 9th, 1876. All reputable practitioners are invited to be present.

L. D. CARPENTER,

*Corresponding Secretary, pro tem.*

### TO READERS AND CORRESPONDENTS.

Communications intended for publication, and exchange journals and papers, should be addressed to the EDITOR OF THE AMERICAN JOURNAL OF DENTAL SCIENCE, 259 N. Eutaw St., Baltimore, Md. All letters relating to the business of the journal, or containing cash remittances, to be directed to Messrs. SNOWDEN & COWMAN. Articles for publication should be received by the editor at least three weeks previous to the first day of the month on which the number in which it is designed for them to appear, is issued.

### CONTENTS:

### ORIGINAL COMMUNICATIONS.

ARTICLE I.—Notes on the American Dental Association, 1875. By S. P. Cutler, M. D., D. D. S. .......... 1

ARTICLE II.—Artistic Knowledge and Critical Taste in Dentistry. By George H. Perine, D. D. S. .......... 7

ARTICLE III—Dental Education. By. W. C. Barrett, D. D. S. .......... 12

Georgia State Dental Society .......... 17

### SELECTED ARTICLES.

The Food we Eat .......... 39

A Dangerous Dentist .......... 41

### MONTHLY SUMMARY.

Chloral as an Anæsthetic .......... 42

Painless Opening of Abscesses .......... 42

The Blue Line in Lead Poisoning .......... 43

Resection of a Portion of Lower Jaw .......... 43

Topical Application in Painful Dentition .......... 44

Milk as a Vehicle of Contagion .......... 44

Avoidance of Phosphorous Poisoning .......... 45

Borax as an Antiseptic .......... 45

Necrosis of the Lower Jaw .......... 45

The Discovery of Anæsthesia .......... 46

To Check Epistaxis .......... 46

Female Students .......... 46

Medicated Ice .......... 47

Extracting Teeth for Brain Disease .......... 47

The Plethysmograph .......... 48

For Burns .......... 48

Cosmoline .......... 48

☞ The American Journal of Dental Science will contain fifty pages of reading matter in each number, making a volume of not less than

*Six Hundred* pages

EXCLUSIVE OF ADVERTISEMENTS.

THE

### AMERICAN JOURNAL

OF

### DENTAL SCIENCE.

**F. J. S. GORGAS, M. D., D. D. S., Editor.**

The *Journal* is issued on the first of each month and contains not less than *fifty* pages of reading matter in each number, exclusive of advertisements, making a volume of *six hundred* pages per annum.

In each number will be found Original Communications, Correspondence, Selected Articles, Monthly Summary, Bibliographical Notices and Editorial Matter, and every effort will be exerted to render the *Journal* worthy of the patronage of the Dental profession.

A generous co-operation is respectfully solicited from the members of the profession; and as the *Journal* has now a printing office of its own, which materially lessens the cost of its publication, it will be issued at the reduced subscription price of

**$2.50 Per Annum in Advance.**

Communications are respectfully solicited on all subjects pertaining to the practice of dentistry, as well as reports of cases occurring in dental practice.

### RATES OF ADVERTISING.

| | 1 MO. | 2 MO. | 3 MO. | 6 MO. | 12 MO. |
|---|---|---|---|---|---|
| 1 PAGE. | $15 00 | $22 50 | $30 00 | $50 00 | $100 00 |
| ½ " | 10 00 | 15 00 | 20 00 | 30 00 | 50 00 |
| ¼ " | 7 00 | 11 00 | 15 00 | 20 00 | 30 00 |
| ⅛ " | 5 00 | 8 00 | 11 00 | 15 00 | 20 00 |

All original communications designed for publication, works for review, new instruments for notice, exchanges, &c., should be directed to the editor, 259 N. Eutaw St., Baltimore, Md.

All advertisements and subscriptions should be addressed to

SNOWDEN & COWMAN,

*Publishers of the Am. Journal of Dental Science.*

86 W. FAYETTE ST. BET. CHARLES & LIBERTY STS.,

BALTIMORE, MD.

